# Reimagining the Clinical Competency Committee to Enhance Education and Prepare for Competency-Based Time-Variable Advancement

**DOI:** 10.1007/s11606-022-07515-3

**Published:** 2022-04-20

**Authors:** Mary Ellen J. Goldhamer, Maria Martinez-Lage, W. Stephen Black-Schaffer, Jennifer T. Huang, John Patrick T. Co, Debra F. Weinstein, Martin V. Pusic

**Affiliations:** 1 Massachusetts General Hospital, Boston, MA 02114 USA; 2 Mass General Brigham, Boston, MA USA; 3 Harvard Medical School, Boston, MA USA; 4 Boston Children’s Hospital, Boston, MA USA; 5 University of Michigan Medical School, Ann Arbor, MI USA; 6 Michigan Medicine, Ann Arbor, MI USA

**Keywords:** competency-based medical education, time-variable graduate medical education, competency-based advancement, clinical competency committee, Milestones, individualized learning plan, COVID-19

## Abstract

Assessing residents and clinical fellows is a high-stakes activity. Effective assessment is important throughout training so that identified areas of strength and weakness can guide educational planning to optimize outcomes. Assessment has historically been underemphasized although medical education oversight organizations have strengthened requirements in recent years. Growing acceptance of competency-based medical education and its logical extension to competency-based time-variable (CB-TV) graduate medical education (GME) further highlights the importance of implementing effective evidence-based approaches to assessment. The Clinical Competency Committee (CCC) has emerged as a key programmatic structure in graduate medical education. In the context of launching a multi-specialty pilot of CB-TV GME in our health system, we have examined several program’s CCC processes and reviewed the relevant literature to propose enhancements to CCCs. We recommend that all CCCs fulfill three core goals, regularly applied to *every* GME trainee: (1) discern and describe the resident’s developmental status to individualize education, (2) determine readiness for unsupervised practice, and (3) foster self-assessment ability. We integrate the literature and observations from GME program CCCs in our institutions to evaluate how current CCC processes support or undermine these goals. Obstacles and key enablers are identified. Finally, we recommend ways to achieve the stated goals, including the following: (1) assess and promote the development of competency in all trainees, not just outliers, through a shared model of assessment and competency-based advancement; (2) strengthen CCC assessment processes to determine trainee readiness for independent practice; and (3) promote trainee reflection and informed self-assessment. The importance of coaching for competency, robust workplace-based assessments, feedback, and co-production of individualized learning plans are emphasized. Individual programs and their CCCs must strengthen assessment tools and frameworks to realize the potential of competency-oriented education.

## Vignette:

*Leila is in her second year of internal medicine (IM) residency. Before emigrating to the United States (U.S.), she had completed IM training in her native country and practiced independently for 2 years.*

*There was little discussion of Leila at the Clinical Competency Committee’s (CCC’s) regular 6-month meeting: her evaluations consistently reflected “exceeding expectations.” When Leila met with her residency program director, no specific summative information was available from the CCC. The available assessment data was generic and interpreted by the program director as “doing fine.” Leila left the meeting wondering about the CCC’s role, and how it helps optimize her educational trajectory. Leila also questions why she needs to finish 3 years of residency, since she was a practicing doctor prior to emigrating to the U.S.A., and all evaluators note her advanced skills. Leila’s program is participating in a competency-based time-variable GME pilot, where advancement is based on demonstrated competency rather than time in training. How can the CCC utilize available assessments to determine Leila’s readiness for unsupervised practice?*

## INTRODUCTION

Assessing physicians-in-training is a high-stakes activity. Effective assessment is important throughout training so that identified areas of strength and weakness can guide educational planning to optimize outcomes. Then, as residents and fellows complete their training, assessment provides the basis to confirm competence for unsupervised practice. Periodic assessment during graduate medical education (GME) should also help physicians-in-training hone their ability to *self*-assess and regulate their learning^1^—critical skills and a career-long responsibility essential for high-quality patient care which can be cultivated through informed self-assessment.^2^

Recognizing the importance (and historic under-emphasis) of assessment, medical education oversight organizations such as the Accreditation Council for Graduate Medical Education (ACGME) have strengthened related requirements in recent years.^3–5^ Growing acceptance of competency-based medical education (CBME), and its logical extension to competency-based, time-variable (CB-TV) GME, highlights the importance of implementing effective, evidence-based approaches to assessment.^6–8^ The implementation of CCCs in the USA, and their equivalent in Canada, Switzerland, the Netherlands, and globally through ACGME-International accreditation, is an outgrowth of widespread educational reform promoting a reorientation of trainee assessment.^4,6,9–16^ In addition, COVID-19’s disruption to routine residency and fellowship training amplifies the importance and urgency of having sound and trustworthy assessment processes to determine readiness for advancement.^17–23^

Clinical competency committees (CCCs) are the lynchpin of assessment in GME—the locus for interpreting evaluative information and determining further actions. When the ACGME initiated its requirement to implement CCCs as part of the “Next Accreditation System,” the committees’ key responsibilities were outlined, with the details of implementation left to each program’s judgment.^4,5^ Varying approaches have now been described in the literature, and the third edition of a CCC guidebook for GME programs was issued by ACGME in 2020; however, a clear best approach has yet to be identified.^24^

Studies have sought to evaluate CCC structure, process, composition, and outcomes^25,26^; correlation of faculty ratings with trainee self-assessment^27–29^; the role of competency coaches^30^; and trainee ability to develop meaningful individualized learning plans (ILPs).^31^ Other studies have sought to elucidate how trainees in internal medicine, pediatrics, emergency medicine, visual diagnostic, surgical, and procedural specialties^7,14,27,32–39^ are assessed on the specialty-specific Milestones and Entrustable Professional Activities (EPAs)—which is essential for competency-based advancement decisions.^22,39^ Additional studies have evaluated the impact of CCC competency decisions on subsequent levels of supervision and independence during residency training.^7,27,35,36^

At Mass General Brigham, the participation of several residency programs in a CB-TV GME pilot^40^ (e.g., where advancement and graduation are based on demonstrated competency rather than solely on time spent in a program) has stimulated closer examination of CCC processes in order to enhance their effectiveness and ensure trustworthy data-informed decisions about individualized advancement from residency to unsupervised practice.^40^ Our engagement with CCCs in several residency programs considering participation in the pilot, along with our review of the CCC literature, has led us to reconceptualize the goals of residency program CCCs and make recommendations for achieving them.

## BACKGROUND

The ACGME’s “Next Accreditation System” and Milestones project call for residency programs to assess the developmental progression of each trainee in terms of measurable competencies, reflecting widespread consensus favoring a competency-based framework for medical education.^4^ CCCs are the principal vehicle for synthesizing available data to assess trainee performance and, importantly, developmental progression over time.^4,24,41,42^

## CCC Goals

The ACGME’s “Common Program Requirements” outline the following core responsibilities of the CCC: (1) review all resident evaluations at least semi-annually; (2) determine each resident’s progress on achievement of the specialty-specific Milestones; and (3) meet prior to the residents’ semi-annual evaluations and advise the program director regarding each resident’s progress.^5^ The ACGME’s “Clinical Competency Committees: A Guidebook for Programs” delineates (Table 1, p 5–7) 41 granular items as “purposes” of the CCC, organized by stakeholder groups (“the program itself, program directors, faculty members, program coordinators, residents and fellows, the institution, and the ACGME”), but notes that “the ultimate purpose is to demonstrate accountability as medical educators to the public: that graduates will provide high quality, safe care to patients while in training, and be well prepared to do so once in practice.”^24^

Programs note that ongoing assessment and CCC consideration of every resident requires considerable time and resources.^24^ ^p 18-22^ However, the negative impact of sub-optimal assessment, such as delayed recognition of competency gaps, can cost considerably more. Moreover, if an opaque, under-resourced assessment system results in failing to maximize individual potential, and perhaps even allows less-than-competent trainees to graduate, the downstream costs to society are far greater. For these reasons, it is essential that GME programs strengthen the developmental assessment of all trainees to improve education today and prepare us for time-variable graduation based on demonstrated competency as a model for the future.

We propose that CCCs have three core goals. First, ***the CCC must regularly and iteratively discern and describe the developmental status of each resident for the purpose of optimizing their education***. This requires aggregating and interpreting a variety and sufficient volume of evaluative material—with an emphasis on multi-source (“360-degree”) evaluations, drawn from a sufficient variety of settings and informed by direct observation.^43,44^ It also requires that CCC findings are incorporated in an individualized educational plan, where summative assessments are incorporated into an action plan co-produced with each trainee.^24^

The CCCs’ second goal relates to GME programs’ fundamental responsibility to protect the public by graduating competent physicians. Thus, ***CCCs must affirmatively determine each resident’s readiness for unsupervised practice*** to support graduation decisions. This requires having explicit promotion criteria that can be applied consistently.

We assert that a third key goal of CCCs is to ***foster each resident’s ability to take responsibility for their ongoing learning, the collection of skills variably known as self-assessment, self-monitoring, and self-regulation of learning***.^1,2^ Understanding one’s own level of skill, knowledge, and judgment is central to providing good care. An important tenet of CBME is the shift of learning control from the faculty to the resident. ^6,45,46^ Physicians must discern when to seek help in delivering care; when to pursue additional education, training, or practice (e.g., simulation); or when to limit their scope of practice—rather than relying on external, usually post hoc oversight of their independent practice. The ability cannot be assumed to develop spontaneously; in fact, studies have demonstrated that highly competent physicians tend to under-rate themselves while the less competent overrate themselves.^47^ Thus, informed self-assessment is a relevant skill to cultivate and ensure during training, linked to the CCC process.^2,24^ The importance of self-assessment and reflective practice is underscored by the recent implementation of the harmonized ACGME Milestone 2.0 sub-competency, “Practice-based Learning and Improvement-2”—“Reflective Practice and Commitment to Personal Growth.”^1,48^

## How Do CCCs Fare in Fulfilling These Goals?

### Formative and Summative Workplace-Based Assessments Inform CCC Decisions

While the ACGME Common Program Requirements and CCC Guidebook provide a framework for CCCs, some evidence indicates that CCCs fall short of meeting these requirements in adequately evaluating the developmental trajectory of trainees.^5,24,25,49–53^ The inception of the ACGME Outcomes Project in 2001 established the six core competencies and stimulated the competency-based medical education movement in the USA, defining the roadmap for GME training outcomes.^3^ Since that time, the ACGME has recommended both formative and summative assessment methods to evaluate trainees. Examples of formative assessment methods include competency-based multi-source evaluation (e.g., evaluation of trainees by faculty, peers, patients, other healthcare professionals, and self-assessment), direct observation with feedback, objective structured clinical examinations, and chart review.^5,24,43^ Summative trainee assessment was then strengthened by the implementation of bi-annual evaluation on specialty-specific Milestones as part of the “Next Accreditation System” in 2013.^4^ Pediatrics has used individualized learning plans (ILPs) for more than a decade, and co-production of ILPs with program leadership is a recent requirement for trainees in all specialties.^5,54,55^ The requirement for both formative and summative assessment has led to innovation and collaboration among academic centers to understand how trainees can be assessed across the continuum of learning and how competency-based assessment supports competency-based medical education.^7,56^ ACGME assessment requirements have stimulated CCCs to codify a process and timetable for evaluations, to collect a sufficient number of evaluations [though what number of evaluations suffices remains subjective], and to incorporate multiple perspectives, including from members with first-hand experience working with residents.^56–58^ With the movement to competency-based medical education and consideration of competency-based advancement, Kinnear and others have described a validity argument for how workplace-based assessment and the CCC process can support competency-based advancement.^8,59^

At the same time, however, in several ways, CCCs are failing to support—and sometimes distinctly undermining—the three stated goals.^51,53,60^ Table 1 outlines current obstacles and key enablers to achieving the three CCC goals. We will explore these obstacles and highlight three recommended “focus areas” for CCCs as they aim to meet the proposed goals and enhance competency-based assessment decisions.

**Table 1:** Current Obstacles and Key Enablers to Advancing the CCC Towards Competency-Based Advancement and Competency-Based Time-Variable Promotion Decisions

CCC goal	Current landscape	Specific limitations	Proposed improvements	Examples for vignette
Discern and describe the developmental status of each resident to optimize education	Lack of a shared mental model of how to conduct trainee developmental assessment^50^	Straight-line scoring on the Milestones	Provide faculty development activities aimed at a shared model of assessment and competency-based advancement. CCCs synthesize evaluative feedback for all trainees, whether struggling, average, or exceptional (like Leila), to inform individualized learning plans, co-produced by trainees with program leadership	Data is available that takes into account Leila’s unique journey, allowing individualization
	Lack of a shared mental model of how to conduct trainee developmental assessment^50^	Focus on outlier identification	Discuss EVERY trainee at the CCC meeting with a view to providing forward-oriented recommendations, based on the competency model.	Developmental perspective allows Leila to plan and adjust her training experiences; educational value becomes a criterion for activity scheduling
	Failure to address coach-evaluator tension	CCC members often fill both coach and evaluator roles	Diversify CCC membership to include a wide range of stakeholders, including those who do not necessarily have an education role	Clear separation of coach and evaluator increases opportunity for Leila to confide stressors and to adopt growth mindset
	CCC may not have sufficient diversity in terms of race, gender, ethnicity, LGBTQ+	Prone to implicit bias and to counter-productive group dynamics	Ensure diversity of CCC membership, explicit consideration of the group processes	Leila was pleased to see a foreign medical graduate represented on the CCC.
Determine each resident’s readiness for unsupervised practice	Lack of explicit competency-based criteria to determine readiness for graduation and unsupervised practice	Advancement is based on demonstration of specific, observable positive behaviors, rather than absence of problems or sanctions	Utilize explicit criteria for competency-based advancement including achievement of the ACGME Milestones	Leila understands what competencies she needs to demonstrate in order to graduate, and where this has or has not been accomplished
Foster each resident’s ability to self-assess	Resident self-evaluation and reflection often only done informally	Informed self-assessment, self-monitoring, and reflective practice are underemphasized by faculty and undervalued by trainees	Ensure that residents practice the skills of informed self-assessment. Incorporate trainee Milestone self-assessment into CCC meeting discussion Utilizing CCC determinations for co-produced individualized learning plans	As Leila learns to self-assess, she understands in which areas she is less strong than others and understands what additional growth is needed to graduate
	Few data visualizations available, and even fewer that are informed by a competency model	When examinations are the key data point, that sends a message as to what is valued	Adopt a quality improvement mindset for self-improvement, where data visualizations play a key role	Leila works with her program director to make evidence-based decisions to determine which elective rotations or other experiences will enable her to achieve competency

### Key Obstacles and Recommended Areas of Focus to Achieve CCC Goals

**Focus Area #1:** ***Assess and promote the development of competency in all trainees, not just outliers, through a shared model of assessment and competency-based advancement***^50,56^

*The CCC should review and synthesize all assessments that inform each trainees’ developmental trajectory towards achievement of competency and provide this information to trainees.* ***Trainees can then use determinations and feedback from the CCC to co-produce an individualized learning plan with program leadership during bi-annual meetings, potentially with participation of a coach.***^24^ ^p. 44-45,50^

Many CCCs, especially those with large numbers of residents, focus primarily on outliers, those few residents who are struggling. Hauer and colleagues evaluated the structure and function of CCCs in 34 residency programs at 5 public institutions in California.^60^ Using semi-structured interviews with program directors, they found the majority of the CCCs had an outlier approach, focusing primarily on struggling trainees rather than using a developmental approach to address the individual needs of *all* trainees.^60^ Schumacher and colleagues developed a structure for identification of the struggling pediatric trainee but noted the need to also develop a process to identify outliers at the other extreme—the exceptional trainee.^36^ While this approach would include more trainees under the CCC’s consideration, it still falls short of a thorough assessment of each individual to provide granular, thematic feedback about their areas of relative strength or weakness to inform ongoing training or refine the self-assessment capabilities.

The failure to individualize all trainee assessments has in some cases led to “straight line scoring,” where all trainees are assigned the same milestone sub-competency score, rather than considering demonstrated competency, undermining the milestone evaluation process.^52,61^ This is compounded when CCCs lack a shared model on CCC process and function; these norms of outlier identification and straight-line scoring become established, and then are hard to break.^50^ In order to ***discern and describe the developmental status of each resident for the purpose of optimizing their education,*** the CCC must first establish a shared model and commitment to reviewing each individual resident and providing summative feedback that can be used by trainees to co-produce an ILP with program leadership.^5,24,50,55,56,58,60,62^ Faculty development for CCC members is essential to mitigate biases that could potentially influence CCC ratings, including bias regarding gender, race, ethnicity, and other forms of cognitive bias.^53,63,64^ CCCs are encouraged to think deliberately about the diversity of their membership and incorporate the science of effective group processes to ensure fair, unbiased committee discussions and decisions.^25,26^

**Focus Area #2:** ***Strengthen CCC assessment and coaching processes for the determination (and promotion) of trainee readiness for independent practice***

The CCC should be structured to explicitly incorporate the useful tension between formative and summative assessment, with workplace-based formative assessment gathered through direct observation, multi-source evaluation and feedback, competency coaching, and summative assessment on the specialty-specific Milestones.^39,65,66^ Coaching is the provision of support and instruction by someone acting as a learner advocate.^67,68^ Coaching provides the opportunity to directly observe trainees and provide specific feedback in an area(s) of competency, moving trainees along the Milestones trajectory towards competence and readiness for independence.

**Figure 1:**
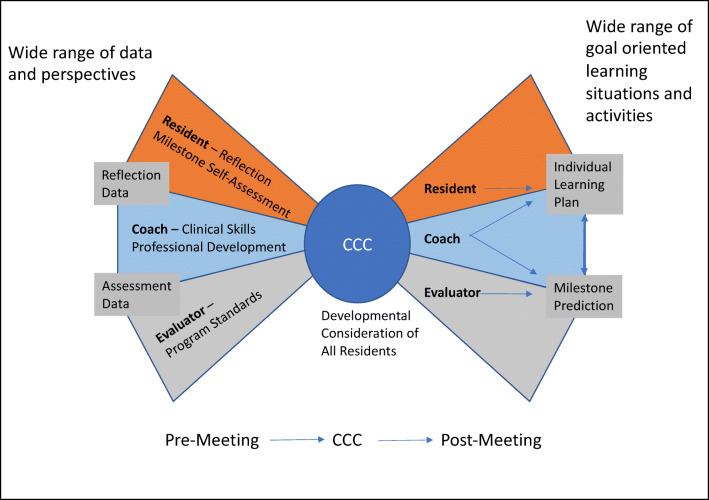
Legend: Bow-Tie Framework for CCC process. A wide range of data are collected and interpreted from three important perspectives: the resident, the coach-advocate, the program advocate. These unique perspectives on the data are kept in balance through data sharing and defined processes validating each perspective. Pre-work leads to an efficient, focused process during the CCC meeting. Conclusions are communicated in the form of both modified learning plans to support the development of each resident and Milestone *predictions* that promote downstream adjustment of the learner’s path.

The majority of coaching programs in both undergraduate and graduate medical education focus on student and trainee career development and wellness while few programs offer coaching that utilizes methods aimed to enhance clinical skills and achieve clinical competence.^30,67–70^ Further, we postulate that insufficient attention is paid to the potential complementarity of formative coaching and summative assessment.^69,71^ The R2C2 [build **r**elationships, explore **r**eactions, explore **c**ontent, and **c**oach for change] model has been validated across specialties and offers specific strategies for both longitudinal and “in-the-moment” coaching focused on patient care, clinical skills, and competency achievement.^67,69,71,72^ Coaching models such as the R2C2 model strive to manage the tension between coaching on the one hand and the need for evaluation on the other, by emphasizing creation of a personal relationship and positive interactions between the coach and resident.^24,30,67,68,73,74^ When coaches serve a dual role of both evaluator and coach on the CCC, this undermines trust and their subsequent ability to serve as a coach.^65,75^ Frequently, the same CCC member provides both a coach and evaluator perspective, not based on design but on coincidental intersection with individual trainees in the clinical environment; we advocate for these roles to be served by different persons who can provide distinct and individualized perspectives.^30,65,75^ The “Bow Tie Framework” delineates the roles and responsibilities of the resident, competency coach, and evaluator in the CCC process (Fig. 1).

**Focus Area #3:** ***Promote informed self-assessment by each trainee to identify learning needs***

*Resident-informed self-assessment should be a celebrated component of the CCC process.*

Despite the growing appreciation for the importance of self-reflection, CCC structures often have under-developed mechanisms for celebrating and encouraging a dialectic between the resident’s developing skill of self-assessment and the recognized standards set forth by each specialty.^28,29,76,77^ Self-regulated learning and professional accountability both depend on recognizing when one needs additional knowledge, enhanced skill, or direct assistance in order to deliver excellent care. Thus, a key prerequisite for independent practice is not only a collection of experience and demonstrated skills but also the ability to recognize gaps and opportunities, especially in regard to continually evolving professional standards.^27,31,78–81^ There is increasing recognition that self-assessment and reflective practice are practiced skills that can be encouraged and incorporated into a program’s culture.^1,2,28,29,31,33,78,81,82^ For example, calls for an increased emphasis on meta-cognition and adaptive expertise explicitly point to the importance of informed self-assessment as well as self-monitoring.^1,83–87^ Discernment, the ability to judge one’s limits, is a key component of entrustability, another increasing emphasis in modern health professions assessment frameworks.^32,88,89^

We suggest that CCCs adopt a standard process of *incorporating* resident Milestone self-evaluation as part of the CCC deliberations instead of having trainees compare their self-determined Milestone ratings to those of the CCC post meeting.^77^ This serves to incorporate the trainee perspective *into* the CCC and ensures the trainee is aware of the trajectory of competence progression in their chosen specialty.^76,77^ CCCs will need to have a mechanism in place to address marked discrepancies, which can and should be discussed during the bi-annual program director-trainee meeting and during the process of co-producing the trainee’s ILP.^24,62^

Further, individualized learning plans offer trainees and program faculty a process to define both short- and long-term goals through a forward-looking lens or roadmap towards competence.^24,54,62^ A study by Li and colleagues found that pediatric residents’ ability to write actionable goals significantly improved over the course of residency training.^31^ Additional studies have focused on coaching and the use of learning change plans, an ILP equivalent.^90^ Under an outlier identification model, CCC data is used to identify and customize the learning plans of only a small number of outliers.^60^ Have problems with this resident been identified? If not, then they can carry on in a standardized program. An individualized approach to assessment and educational planning is taken only if problems are identified. Under a forward-looking, ILP perspective, data are used not only to identify problems, but to map when and how each competency or milestone can be achieved by each resident, helping to chart the best path forward to optimize each learner’s development, including those “ahead of the curve.”^36^ Co-production of an ILP by every resident, based on the input of the CCC, is then used to actualize this objective.^24,54,62,90^

The ILP process leads to finer-grained examination of the existing data in the light of the resident’s remaining scheduled activities, including an emphasis on longitudinal learning trajectories. For programs utilizing competency-based advancement or preparing to pilot CB-TV GME graduation, determining each resident’s appropriate graduation date involves risk and opportunity for both the resident and the program.^7,16,21–23,91–93^ This dynamic can be a positive force for ensuring that data collection and interpretation is transparent and fully codified. Each individual resident’s ILP should include relevant data-driven predictions, creating both short- and long-term actionable goals. We assert that this data-driven ILP process is beneficial to all programs regardless of whether they are piloting a time-variable graduation date.

### Connecting the Goals: Data Management as an Enabling Skill of All Stakeholders

*To accomplish its goals, the CCC must utilize effective mechanisms to collect a wide range of data, analyze both its quality and sufficiency, and develop robust reporting mechanisms. The ACGME CCC Guidebook includes recommendations to manage administrative tasks and defines the roles and responsibilities for each member of the CCC.*^24^ ^p14-16;18-22^ *While all GME programs must utilize robust assessment, time-variable training provides a more urgent stimulus to strengthen assessment, given the necessity of making evidence-based graduation decisions based on demonstrated competency.*^21,22,56,93^

*The following are recommendations to strengthen the CCC process:*

- Hold meetings frequently enough to avoid data overload. More frequent meetings should also help to ensure that rotation-based assessments are completed without many months of delay and can help address concerns in a timely fashion, as well as ensuring that developmental needs are addressed on a timescale consistent with the learning.
- Parse the workload by assigning CCC members a manageable subset of residents whose data they review and report on—or, alternatively a subset of competencies for which they review all resident data. These two perspectives are complementary.
- Utilize multi-source data that incorporate formative and summative assessments, incorporating clinical outcomes data when available.
- Use data visualizations to highlight individual or programmatic trends.^94,95^ The degree to which a CCC can carry out its work without the inside knowledge of the residency program director is a measure of its ability to serve as a complementary check on the day-to-day functioning of the program. An ideal information system to support CCC operation includes a data portfolio that can run the gamut from individual observations, through summations of individual resident achievement, to integrative displays at the program level.

**Figure 2:**
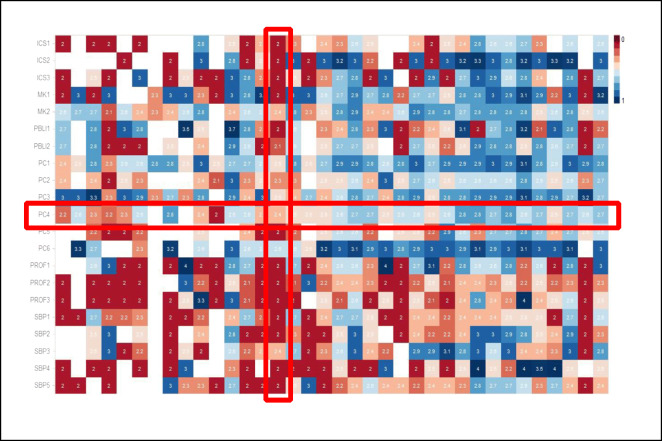
Legend: Heat map visualization of Milestone competency achievement in one program. An integrated heat map from one residency program’s CCC data, utilizing the system’s independent “Passport” system of Milestone competency assessment which evaluates each Milestone sub-competency. Each column represents one resident; each row, one competency; each cell, the cumulative longitudinal consensus of his or her evaluators. The color corresponds to the ranking, with red scores lower on the developmental progression than blue. White squares indicate missing data. While individual residents vary in their ratings, the program overall is likewise more successful in achieving some competency elements than others. The columns are organized with the more junior residents to the left and the more senior resident to the right. The rows correspond to the ACGME Pathology Milestone sub-competencies, based on the six core competencies. ICS1, Interpersonal and Communication Skills sub-competency 1; MK1, Medical Knowledge sub-competency 1; PBL, Practice-Based Learning and Improvement; PC, Patient Care; PROF, Professionalism; SBP, Systems-Based Practice. This heat map incorporates approximately 5600 datapoints. Figure courtesy of Drs. Emilio Madrigal and Long Phi Le, Department of Pathology, Massachusetts General Hospital.

Consider the heat map shown in Figure 2 which can provide a perspective on each of the CCC goals we have described. Each column represents a single resident, and so, the visualization can show all residents in the program. Each row represents a single Milestone sub-competency (or EPA) so that the columns taken together represent the entirety of the competency model for the specialty. Each cell represents how that individual resident is doing on that individual competency, with the temperature of the color suggesting a five-point scale of longitudinal achievement. As such, the representation provides a summary of the current state of the program, with the between-resident variability manifest at a glance, especially if the residents are ordered by stage of training. The variability between competency elements is also on display with their differing rate of achievement. Clearly, some competencies are easier to develop than others. Clearly, some residents are further along in their development than are others. The visualization is consistent with the breadth of the CCC’s mission, across all residents and across the entire competency model. A further embellishment would be to represent resident self-assessment data on the same grid.

Our example is a static visualization. Ideally, CCCs are supported by dynamic dashboards which allow the members to consider multiple views on the data, drilling down when necessary, to the granular data that determine the current estimate of milestone progression.^94–96^ An important point here is that the CCC can assess the sufficiency of the evaluation data available to it. What data is missing? Why is it missing? Are there program-level quality improvement (QI) implications? Or specific implications for this resident? As the locus of control for assessment is tilted towards a self-regulated resident learner, the degree to which the learner is able to meet the program expectations in terms of collecting the necessary evidence of achievement may be its own datapoint. CCC data visualizations should be engineered to allow dynamic access within the CCC meeting to provide both an overall program-level map, and to drill down to the individual data point level.

## Conclusion

In this article, we have proposed three core CCC goals that must be regularly applied to *every* resident: (1) discern and describe developmental status to optimize education, (2) determine readiness for unsupervised practice, and (3) foster self-assessment ability. We have recommended areas of focus to enhance the CCC process to actualize these goals including the following: assess and promote the development of competency in all trainees, not just outliers, through a shared model of assessment and competency-based advancement; strengthen CCC assessment processes to determine trainee readiness for independent practice; and promote informed self-assessment of each trainees’ learning needs. We have emphasized the importance of providing formative feedback through coaching and robust workplace-based multi-source assessments to inform the CCC’s determination of the developmental trajectory of each trainee coupled with co-production of an individualized learning plan. Further, we emphasize the importance of data visualizations to provide a comprehensive overview of each trainee’s competency trajectory, noting areas of both strength and growth.

Institutions and programs must recognize that trainee assessment is a critical and resource-intensive process and must prioritize and fund it accordingly. Participating faculty should be appropriately trained and compensated for their effort.^64^ In addition, engagement in assessment may (and should) contribute to the academic advancement of faculty, providing another important incentive. Successful strategies to support effective assessment should be disseminated*. Competency-based medical education promotes individualized pathways and requires flexible educational systems regardless of whether programs plan for time-variable advancement.*^6,97^

Overall, we are promoting a forward-looking mindset in service of competency-based advancement, one where the question is not “how have you done until now?” but rather “given what we know about you, how can we help optimize your forward trajectory?”. The ACGME has provided the structure and framework for CCCs to actualize these goals, yet individual programs must conceptualize and strengthen the tools and personalize the framework to realize the potential of the CCC in fulfilling its role in competency-based medical education and advancement.
